# Galileo: The Added Value for Integrity in Harsh Environments

**DOI:** 10.3390/s16010111

**Published:** 2016-01-16

**Authors:** Daniele Borio, Ciro Gioia

**Affiliations:** 1 European Commission, Joint Research Centre (JRC), Institute for the Protection and Security of the Citizen (IPSC), Security Technology Assessment Unit, Via Enrico Fermi 2749, 21027 Ispra (VA), Italy; 2 Piksel Ltd Italian Branch, Via Ernesto Breda 176, 20126 Milano (MI), Italy

**Keywords:** Galileo, multi-constellation, RAIM, protection levels, integrity

## Abstract

A global navigation satellite system (GNSS)-based navigation is a challenging task in a signal-degraded environments where GNSS signals are distorted by multipath and attenuated by fading effects: the navigation solution may be inaccurate or unavailable. A possible approach to improve accuracy and availability is the joint use of measurements from different GNSSs and quality check algorithms; this approach is investigated here using live GPS and Galileo signals. A modified receiver autonomous integrity monitoring (RAIM) algorithm, including geometry and separability checks, is proposed to detect and exclude erroneous measurements: the multi-constellation approach provides redundant measurements, and RAIM exploits them to exclude distorted observations. The synergy between combined GPS/Galileo navigation and RAIM is analyzed using live data; the performance is compared to the accuracy and availability of a GPS-only solution. The tests performed demonstrate that the methods developed are effective techniques for GNSS-based navigation in signal-degraded environments. The joint use of the multi-constellation approach and of modified RAIM algorithms improves the performance of the navigation system in terms of both accuracy and availability.

## 1. Introduction

Geolocation, *i.e.*, the process of identifying the position of a given object, is present in everyday life; for example, mobile devices, be they smart phones or tablets, are generally equipped with a GPS receiver and cameras, so a user can geo-localize a picture taken with the device. This is only one of the applications classified as location-based service (LBS). Hence, there is a growing request for LBSs that can be carried out in different types of environments, from “open-sky” to indoors; hence, the navigation block has to be able to provide a seamless reliable navigation solution in different scenarios, such as open-sky, open-pit mines, urban canyons or even indoors. These scenarios are characterized by different signal availability and propagation conditions; in the first case, open-sky, a single GNSS is able to provide reliable, accurate and continuous navigation solutions with an accuracy of usually less than 10 m [1]. The good performance and the ease of operations of GNSS navigation in open-sky conditions have stimulated the need of extending GNSS-based navigation in signal-degraded environments, such as open-pit mines, urban canyons and indoors. These scenarios are characterized by a reduced availability of GNSS signals. Moreover, even when the minimum number of signals required to compute the navigation solution is available, gross errors due to multipath and fading effects can degrade GNSS observables. Signal availability and challenging propagation conditions are just two of the major problems that have to be solved in such scenarios: an increased number of satellites and suitable processing techniques are required to enable seamless navigation.

Availability, accuracy, continuity, coverage and integrity [2] are the parameters commonly used to assess the performance of navigation systems. Navigation is enabled only if minimum requirements in terms of these metrics are met. However, GNSS-based navigation may provide unreliable navigation solutions in harsh environments, due to the poor signal quality. Furthermore, in degraded scenarios, the performance requirements may not be satisfied using a single GNSS. For these reasons, several technologies have been proposed to extend LBSs in such environments.

Among the proposed solutions, the multi-constellation approach, *i.e.*, the joint use of measurements provided by several GNSSs, has the potential to enable GNSS usage in environments where navigation using a single GNSS is usually precluded [3,4,5,6,7].

Multi-constellation receivers are able to process an increased number of satellite signals and, thus, can provide a more continuous solution with respect to single constellation devices [3].

Hence, the solution availability (SA) can be significantly improved. Despite these advantages, signal availability is not sufficient to guarantee an accurate navigation solution. Measurements can be affected by gross errors induced by signal attenuation, multipath and fading effects. In this case, the redundancy provided by the multi-constellation approach has to be exploited to isolate and exclude distorted observations. Redundancy is essential for measurement consistency checks [8]; more measurements and stronger geometry conditions enhance the capabilities of quality monitoring procedures, for both detection and identification purposes [9].

Quality monitoring procedures are part of the integrity process, which refers to the ability of a system to provide timely warnings to users when the system should not be used [10]. In GNSS, integrity information can be obtained from different sources.

First of all, GNSSs provide integrity information via the navigation message. This information usually refers to anomalies related to the system and satellite operations, such are satellite clock errors. The integrity information, provided by GNSSs themselves, cannot be used for real-time applications: because the ground control segments can take up to a few hours to identify and report the failure of a satellite [1].

Therefore, additional sources have to be used to obtain the integrity information. Examples of such sources are aircraft autonomous integrity monitoring (AAIM), ground-based augmentation system (GBAS), satellite-based augmentation system (SBAS) and RAIM [1].

AAIM systems use information provided by sensors mounted on-board an aircraft to obtain integrity information [8]; whereas GBAS and SBAS systems compute integrity information exploiting a network of ground stations and satellite constellations, respectively [10]. In the GBAS and SBAS cases, additional infrastructures are required, increasing the complexity and the overall cost of the navigation system. However, errors due to local effects, such as multipath and fading, are not identified using the above-mentioned techniques.

These limitations promoted the development of RAIM, which is considered here: this approach can be the key enabler for error isolation and exclusion. RAIM is a technique that exploits only the information available using a GNSS receiver; RAIM algorithms may detect user-level errors [1]. Such techniques are used for quality monitoring to identify, and eventually exclude, observables affected by gross errors [11].

RAIM algorithms were originally conceived of as part of air navigation systems where they are adopted for safety-critical applications. The classical algorithms, considering only GPS, assume only one measurement failure at a time. In harsh environments, such an assumption may not be sufficient, since often, two or more outliers can be present in the measurement set. In order to overcome the limitations of traditional RAIM algorithms, different strategies have been proposed using multi-constellation solutions. Moreover, in harsh environments, the most stringent assumptions is related to the presence of a single blunder in the measurements set. Hence, modified algorithms have to be developed to take into account the presence of multiple blunders and to overcome the limitations of the classical RAIM algorithms.

The RAIM algorithm proposed in this research is a modified version of the classical forward backward (FB) [12] approach and considers a GPS/Galileo multi-constellation scenario. The proposed algorithm provides improved performance with respect to the classical FB algorithm exploiting the introduction of a preliminary check to verify the geometry conditions. Such a test has been adopted since the capabilities of detecting outliers is highly dependent on the measurement redundancy. Moreover, the presence of an outlier can cause the rejection of good measurements; hence, in order to avoid the rejection of blunder-free observables, a separability check is introduced. The fault detection and exclusion (FDE) algorithm implemented is made up of two phases: the forward and backward phases. The first one uses four tests, specifically a preliminary check based on geometry considerations, a global test (GT) to check the consistency of the measurement set [12,13], a local test (LT) to identify the outlier [14] and, finally, a separability test to check the correlation between the observables [15]. These tests will be described in Section 3. The backward phase, where the erroneously-rejected measurements are re-introduced, is based only on a GT test. A complete description of the algorithm is provided in Section 3.

In order to validate the proposed algorithm, several hours of GPS and Galileo data were collected in a signal degraded environment where a multi-constellation receiver was placed between high reflecting buildings. The performance of the GPS/Galileo multi-constellation solution is assessed and compared with respect to that obtained using only GPS. The improvements achieved using the proposed RAIM algorithm are assessed. The navigation system performance is evaluated using three parameters, specifically standard deviation (SD), mean and maximum errors. These parameters are computed for the horizontal and vertical components of the position and velocity solutions.

The remainder of this paper is organized as follows: the multi-constellation approach along with the estimation techniques adopted are described in Section 2. The RAIM algorithm implemented is introduced in Section 3, and the set-up adopted for the experimental analysis is described in Section 4. Experimental results are analyzed in Section 5, and finally, conclusions are provided in Section 6.

## 2. Multi-Constellation Solution

The position velocity time (PVT) solution is computed using pseudorange and Doppler shift measurements. Specifically, the position of the receiver and its clock bias are estimated using pseudoranges, whereas Doppler shift measurements are exploited to estimate the receiver velocity and the receiver clock drift.

A pseudorange can be expressed as [10]:

(1) $$\rho =d+cdt_{s}-cdt_{r}+e_{orbital}+d_{Iono}+d_{Tropo}+\epsilon_{\rho }$$

where *d* is satellite-receiver distance and $cdt_{r}$ and $cdt_{s}$ are the receiver and satellite clock errors, respectively. $e_{orbital}$ is the satellite orbital error, and $d_{Iono}$ and $d_{Tropo}$ model the ionospheric and tropospheric effects. Finally, $\epsilon_{\rho }$ accounts for unmodeled effects, such as receiver noise and residual errors. Model (1) is not linear in the user position; hence, its solution requires an iterative approach where linearization is performed at each iteration. At each iteration, the system unknowns are estimated and used to predict pseudorange measurements. Differences between actual and predicted measurements, *z*, are formed and related to errors in the system unknowns using a linearized version of Equation (1) [1]. The errors in the unknowns are used to updated the position solution.

After linearization, the pseudorange equations can be written as:

(2) $$\delta \rho =H\Delta x+\epsilon_{\rho }$$

where δρ contains the differences between actual and predicted measurements, Δx contains the unknowns, *H* is the design matrix [1] and, finally, $\epsilon_{\rho }$ contains the residual errors.

GPS and Galileo adopt different time scales, hence when GPS and Galileo observables are used together, the clock inter-system bias has to be added in the navigation solution [7]. Thus, Δx assumes the following form:

(3) $$\Delta x={\left[\Delta \phi ,\Delta \lambda ,\Delta h,\Delta cdt_{r},\Delta cdt_{Sys}\right]}^{T}$$

where Δϕ,Δλ,Δh are the receiver coordinate differences, expressed in the WGS84 frame. These differences are added to the nominal state, $x_{0}={\left[\phi_{0},\lambda_{0},h_{0},cdt_{r,0},cdt_{Sys,0}\right]}^{T}$, during the iterative approach used to solve for the navigation solution. $cdt_{Sys}$ is the inter-system bias. Although the implementation selected is in the WGS84 frame, the algorithm can be implemented in different reference frames using the transformations described, for example, in [16].

The design matrix is defined as:

(4) $$H=\left[\begin{array}{ccccc} a_{GPS_{1}} & b_{GPS_{1}} & c_{GPS_{1}} & 1 & 0\\ a_{GPS_{2}} & b_{GPS_{2}} & c_{GPS_{2}} & 1 & 0\\ \vdots & \vdots & \vdots & \vdots & \vdots \\ a_{Gal} & b_{Gal} & c_{Gal} & 1 & 1 \end{array}\right]$$

where a,b,c are the directional cosines of the vector from the receiver to the satellite positions.

In this paper, a weighted least squares (WLS) estimator is used, where a weighting matrix, *W*, is introduced. The matrix is used to account for the different signal qualities and, in this case, is a function of the satellite elevation [17]. Thus, an estimate for Δx is obtained as:

(5) $$\Delta \hat{x}={\left(H^{T}WH\right)}^{-1}H^{T}W\delta \rho$$

Finally, the position solution is updated as follows:

(6) $$\begin{array}{cc} & x=x_{0}+\Delta \hat{x}\\ & x_{0}=x \end{array}$$

The estimation method used is based on the minimization of the residuals:

(7) $$r=z-H\cdot \Delta \hat{x}$$

The position obtained from the pseudoranges is used together with the Doppler shift measurements, Δf, to compute the velocity of the receiver. Hence, assuming known the receiver coordinates, the receiver velocity can be estimated using the approach described below. A Doppler measurement is a scaled version of the receiver-to-satellite range-rate affected by the receiver and satellite clock drifts. In particular, pseudorange-rates are obtained as [1]:

(8) $$\dot{\rho }=-\lambda \Delta f$$

where *λ* is the signal wavelength. Pseudorange-rates can be modeled as:

(9) $$\dot{\rho }=\dot{d}+c\dot{dt_{s}}+c\dot{dt_{r}}+\epsilon_{\dot{\rho }}$$

where $\dot{d}$ is the geometric range-rate and $c\dot{dt_{s}}$ and $c\dot{dt_{r}}$ are the satellite and receiver clock drifts, respectively. $\epsilon_{\dot{\rho }}$ contains all of the unmodeled errors.

Correcting for the satellite clock drift and replacing $\dot{d}$ with the dot product $(v_{s}-v_{r})\cdot e$ yields:

(10) $$\begin{array}{cc} & \dot{\rho }=\left(v_{s}-v_{r}\right)\cdot e+c\dot{\delta t_{r}}\\ & \dot{\rho }-v_{s}\cdot e=-v_{r}\cdot e+c\dot{\delta t_{r}} \end{array}$$

where e is the unit vector pointing along the line-of-sight from the user to the satellite. $v_{s}$ and $v_{r}$ are the satellite and receiver velocity vectors, respectively.

Considering $\delta \dot{\rho }$ = $\dot{\rho }-v_{s}\cdot e$, the difference between measured and predicted pseudorange-rates is found:

(11) $$\delta \dot{\rho }=-v_{r}\cdot e+c\dot{\delta t_{r}}$$

Expanding the dot product in Equation (11) and considering *k* measurements, a system of equations expressed in matrix form is found:

(12) $$\delta \dot{\rho }=Hv+\epsilon_{\dot{\rho }}$$

where v is the state vector defined as:

(13) $$v=\left[V_{e},V_{n},V_{u},c\dot{\delta t_{r}},c\dot{\delta t_{Sys}}\right]$$

$V_{e}$, $V_{n}$ and $V_{u}$ are the components of the receiver velocity expressed in the east north up (ENU) frame, and $c\dot{\delta t_{Sys}}$ is the inter-system clock drift.

Finally, the user velocity and receiver clock drift can be estimated as:

(14) $$v={\left(H^{T}W_{\dot{\rho }}H\right)}^{-1}H^{T}W_{\dot{\rho }}\delta \dot{\rho }$$

where *H* is the design matrix detailed in Equation (4) and $W_{\dot{\rho }}$ represents the different accuracies of the pseudorange-rates.

## 3. Integrity Algorithm

In hostile environments, the limited number of observables and the presence of multiple large outliers make the design of the quality monitoring algorithm a challenge. The effectiveness of RAIM algorithms is usually assessed considering reliability and separability. The first one is related to the detection capability of the algorithm, whereas the second one is related to the capability to properly identify an outlier and represents the risk of incorrectly flagging a “good” measurement as an outlier. In the RAIM standard [18], for example, the integrity requirements are quite demanding, since both the required detection probability and false alarm rate have to be met for any location and time.

### 3.1. Preliminary Check

Classical RAIM algorithms are applied without a check on the satellite geometry: in signal-degraded scenarios, this can cause a reduction of the performance of the integrity algorithm. For example, in the case of large dilution of precision (DOP), the navigation accuracy degrades; moreover, unfavorable geometry conditions may degrade the performance of the RAIM algorithms. In order to verify the geometric condition, a check has to be performed before the quality monitoring algorithm. This test has to screen out bad geometries, which could imply erroneous detections. With this in mind, a preliminary check has been introduced before the application of the FB technique; the test that screens out bad geometries [10,19] is based on the weighted approximate radial-error protected (WARP) defined as:

(15) $$WARP={WSlope}_{max}\cdot T_{G}$$

where $T_{G}$ is a decision threshold, which will be better defined in the following, and $WSlope_{max}$ is the maximum among the computed WSlope [10]. WSlope is defined as [19,20]:

(16) $$WSLOPE_{i}=\frac{\sqrt{A_{1,i}^{2}+A_{2,i}^{2}}}{S_{i,i}}$$

where:

(17) $$A={\left(H^{T}WH\right)}^{-1}H^{T}W$$

and:

(18) $$S=I-H{\left(H^{T}WH\right)}^{-1}H^{T}W$$

The indices in Equation (16) are used to identify a specific element of *A*.

### 3.2. Forward-Backward

Only configurations characterized by strong geometry conditions, *i.e.*, characterized by a WARP lower than a fixed threshold, are then checked using the FB algorithm. After the preliminary check, the FB method is implemented [9,12,17]. In the first section, the forward phase, a GT, a LT and a separability test, are used. In the second phase, the backward step, only the GT is used.

After the preliminary check, the first step of the FB algorithm is the GT. In this case, the test decision statistic, *D*, is a quadratic form of the residuals, r, defined in Equation (7):

(19) $$D=r^{T}Wr$$

Under the assumption that the observation errors are normally distributed, *D* follows a central chi-square distribution with m-n degrees of freedom. *m* is the number of measurements, and *n* is the number of unknowns, that in the multi-constellation case considered here is five, *i.e.*, the number of elements of Equation (3). *D* is compared to the threshold, $T_{G}$, defined as:

(20) $$T_{G}=\chi_{1-\alpha ,\left(m-n\right)}^{2}$$

where $\chi_{1-\alpha ,\left(m-n\right)}^{2}$ is the abscissa corresponding to a probability value 1-α of a $\chi^{2}$ distribution with $\left(m-n\right)$ degrees of freedom. *α* is the target false alarm probability, *i.e.*, the probability of incorrectly accepting a measurement. Note that $T_{G}$ was already used in Equation (15) for the definition of the WARP parameter. The GT is applied to the whole set of measurements: if the measurement set is declared not to be consistent, then an LT has to be carried out to identify outliers. In this case, the standardized residuals are used as decision variables and are defined as:

(21) $$w_{i}=\left|\frac{r_{i}}{\sqrt{{\left(C_{r}\right)}_{ii}}}\right|\;i=1,\cdots ,m$$

where ${\left(C_{r}\right)}_{ii}$ is the *i*-th diagonal element of the residual covariance matrix $C_{r}$, which is determined, starting from Equation (7), using the general variance-covariance propagation law [12]. The standardized residuals are assumed to be normally distributed and, thus, are compared to a threshold, $T_{L}$, defined as:

(22) $$T_{L}=N_{\left(1-\alpha /2\right)}$$

where $N_{\left(1-\alpha /2\right)}$ is the inverse cumulative density function (CDF) of a Gaussian random variable computed at 1-α/2. The measurement corresponding to the standardized residual exceeding $T_{L}$ is indicated as an outlier and, thus, should be excluded from the navigation solution computation. Before measurement rejection, a further test is carried out to analyze the correlation among observables. In particular, a large blunder can lead to abnormal residuals also in other measurements, causing erroneous measurement rejection. Hence, a test to verify the separability of the measurements is performed. In this case, the decision variable adopted is the correlation coefficient, $\gamma_{ij}$, of the standardized residuals [15]:

(23) $$\gamma_{ij}=\frac{{\left(C_{r}\right)}_{ij}}{\sqrt{{\left(C_{r}\right)}_{ii}{\left(C_{r}\right)}_{jj}}}$$

The coefficients are compared with respect to a threshold, if there is at least one coefficient exceeding the threshold, the measurement identified as an outlier by the LT is not rejected, and the solution is flagged as unreliable. Only if there is no correlation coefficient exceeding the threshold, the measurement is rejected. The former test, which is not present in the classical FB algorithm, concludes the forward phase. If more than one measurement is rejected, the backward phase is implemented to re-introduce measurements erroneously excluded from the navigation solution.

### 3.3. Improvements with Respect to Classical Algorithms

In harsh environments, such as urban canyon or mountainous areas, the hypothesis of a single outlier is not realistic; hence, the presence of multiple blunders should be considered. In order to identify and reject multiple erroneous measurements, quality monitoring algorithms can be performed iteratively. In this case, the algorithm performs recursive rejection of an observation until no additional outliers are present or the solution cannot be checked due to a geometric limit or due to the correlation among the measurements. In this research, the classical FB algorithm has been modified introducing a preliminary check to verify the robustness of the satellite geometry and a separability check to avoid erroneous rejections. The algorithm has been further enhanced considering different accuracies for GPS and Galileo pseudoranges [6,21]. Moreover, a composite design matrix is used for the GPS/Galileo case. In this case, the presence of an inter-system bias is accounted for [7].

## 4. Experimental Set-up

**Figure 1:**
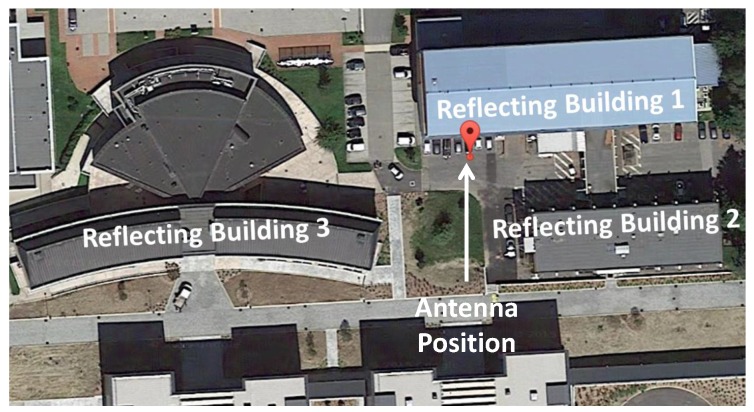
Environment where the antenna was placed for the static test. GNSS signals are degraded by multipath due to the buildings surrounding the receiver.

In order to validate the proposed approach and to analyze the efficiency of the synergy between the implemented RAIM algorithm and the GPS/Galileo multi-constellation solution, static data have been used. The data were collected in the Joint Research Centre (JRC) site in Ispra, Italy. More than two hours of 1-Hz data were collected using a multi-constellation, multi-frequency receiver. The receiver was placed in a parking area between three high buildings, which reflect GNSS signals and cause severe multipath errors in the GNSS measurements. In Figure 1, the environment where the antenna was placed is shown: GNSS signals can be degraded by multipath due to the buildings surrounding the receiver. This type of environment was selected in order to demonstrate the capability of the algorithm to identify and reject multiple outliers.

The receiver used for the test is a Javad Delta 3, which is a multi-frequency, multi-constellation high-precision device. In this study, only L1and E1pseudorange and pseudorange-rate measurements are considered. The Javad receiver was then connected to a geodetic multi-frequency antenna, a GrAnt G5T Javad high performance antenna. The position of the antenna was carefully surveyed in order to obtain an accurate position used as a reference for the computation of the position errors. The coordinates of the antenna are reported in Table 1.

**Table 1:** Coordinates of the antenna placed in a parking area at the Joint Research Centre (JRC) premises in Ispra, Italy.

Latitude (Deg)	Longitude (Deg)	Altitude (m)
45.81121	8.62696	256.466

**Figure 2:**
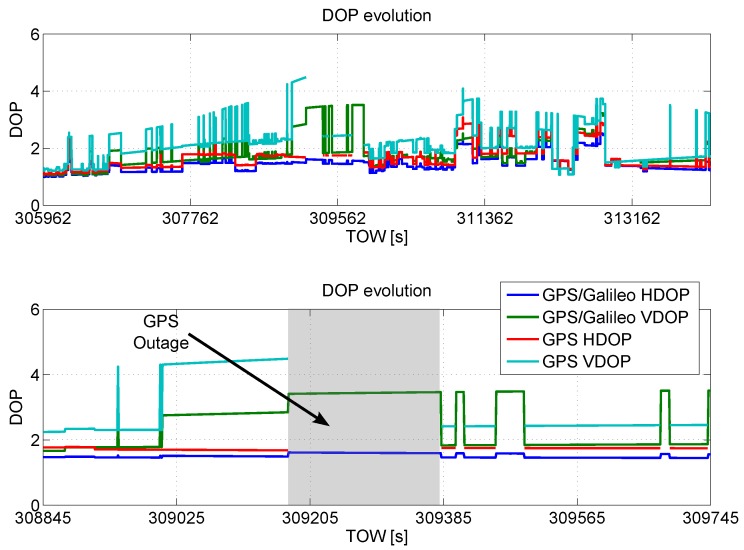
Horizontal dilution of precision (HDOP) and vertical dilution of precision (VDOP) of the configurations without the receiver autonomous integrity monitoring (RAIM) application as a function of time.

In Figure 2, horizontal dilution of precision (HDOP) and vertical dilution of precision (VDOP) of the configurations without RAIM application are plotted as a function of time; from the figure, it emerges that the geometry conditions during the data collection were not so critical. In fact, the HDOP reaches a maximum value of 2.5 and 3.1 for GPS/Galileo and for GPS only, respectively. Only a short outage is present using only GPS, as detailed in the lower box of Figure 2. The inclusion of the Galileo measurements reduces both HDOP and VDOP, and no outages are present in the case of the multi-constellation solution. 

Four different configurations are tested: the configurations are obtained combining GPS and Galileo observations with the RAIM algorithm. Hence, the configurations are:

- The PVT solution computed using GPS only without RAIM (GPS noRAIM)
- The PVT solution computed using GPS and Galileo together without RAIM (GG noRAIM)
- The PVT solution computed using GPS only applying RAIM (GPS RAIM)
- The PVT solution computed using GPS and Galileo together, applying RAIM (GG RAIM).

In order to have a fair comparison among the configurations considered, three time frames are identified:

- All of the available epochs
- Reliable epochs, *i.e.*, only epochs when the solution is declared reliable by the RAIM algorithm
- Reliable common epochs, *i.e.*, the common epochs between the GPS and GPS/Galileo reliable epochs.

## 5. Experimental Results

In this section, the experimental results are presented, and the performance of the configuration considered is analyzed in terms of accuracy and availability. Three parameters are used for the evaluation of the accuracy; specifically, SD, maximum and mean errors. The metrics are computed for horizontal and vertical components of the position and velocity solutions. The continuity of the navigation solution is assessed using two different parameters; specifically, the SA and reliable availability (RA) depending on the application or not of RAIM techniques. The first one is the time percentage when the solution is computed: this parameter is used for the configuration without RAIM; whereas the RA is the percentage of time when the solution is computed and declared reliable: this parameter is used when the RAIM algorithms are performed.

### 5.1. Positioning Results

In this section, the results obtained in the position domain are presented.

**Figure 3:**
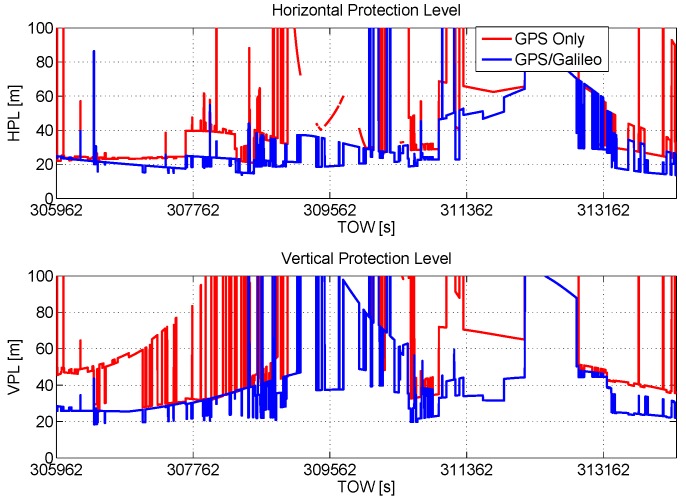
Horizontal protection limit (HPL) and vertical protection limit (VPL) as a function of time. Benefits of the inclusion of Galileo measurements in terms of HPL and VPL.

Two of the outputs of the RAIM algorithm are the horizontal protection limit (HPL) and vertical protection limit (VPL) defined in [1]. In order to demonstrate the benefits of Galileo inclusion, HPL and VPL are plotted as a function of time in the upper and lower boxes of Figure 3. The improvements due to the Galileo inclusion clearly emerge from Figure 3: both HPL and VPL are lower than in the GPS-only case. 

**Figure 4:**
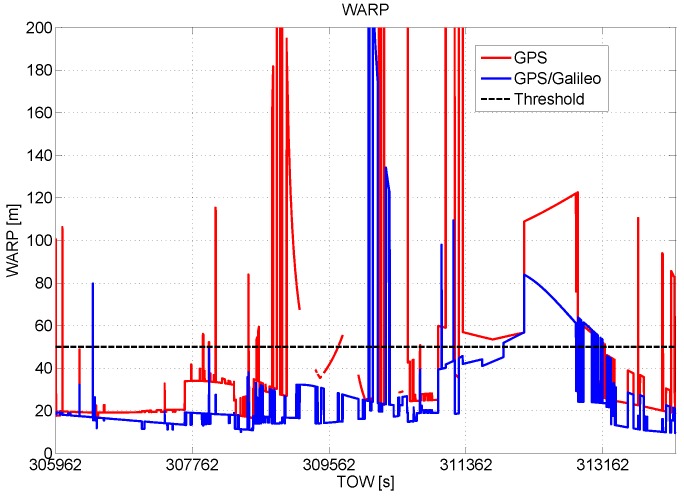
Weighted approximate radial-error protected (WARP) as a function of time. Benefits of the inclusion of Galileo measurements.

In Figure 4, the WARP values are plotted as a function of time and with respect to the threshold, $T_{G}$. The benefits of the introduction of the Galileo observables are evident as for the HPL and VPL cases.

**Figure 5:**
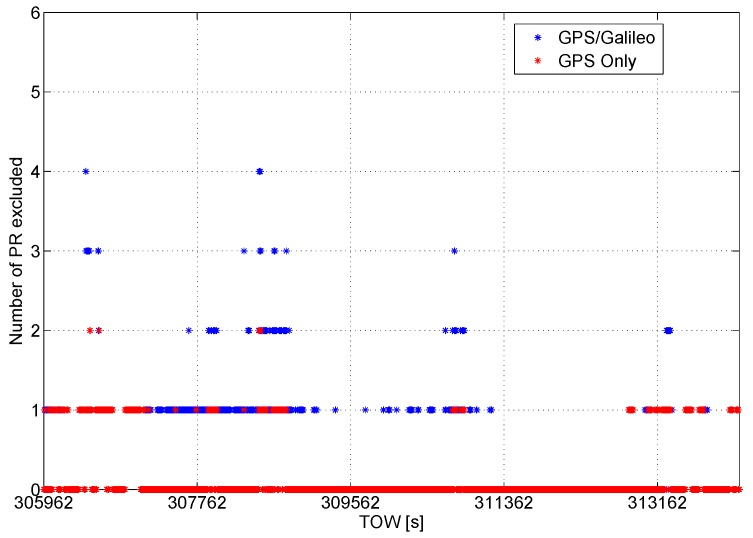
Number of pseudoranges excluded epoch by epoch. More exclusions can be performed when the multi-constellation approach is adopted.

The introduction of the Galileo measurements provides an enhancement of the redundancy of the system; this is a key enabler for the improvements of the performance of the RAIM algorithm. The enhanced redundancy allows the rejection of a higher number of measurements affected by gross errors. The number of pseudoranges excluded epoch by epoch is shown in Figure 5: in the multi-constellation, case a higher number of outliers can be identified and rejected.

The SA for the data collection in the case of GPS alone assumes a high value, about 95%. The inclusion of the Galileo measurements enhances the SA, and almost no outages are present in the multi-constellation solution. The application of the RAIM algorithm reduces the availability of the solution, specifically the RA, that in the GPS-only case is more than halved with respect to the SA. When the RAIM algorithm is applied, the benefit of the inclusion of the Galileo measurements is more evident; the RA in the case of the multi-constellation solution is almost twice that of the single system case and passes from 43% to 78%. The results related to the SA and RA of the position solution are detailed in Table 2.

**Table 2:** Solution availability (SA) and reliable availability (RA) of the different solutions. GG, GPS and Galileo.

Configuration	SA (%)	RA (%)
GPS noRAIM	95	N.A.
GG noRAIM	100	N.A.
GPS RAIM	95	43
GG RAIM	100	78

**Figure 6:**
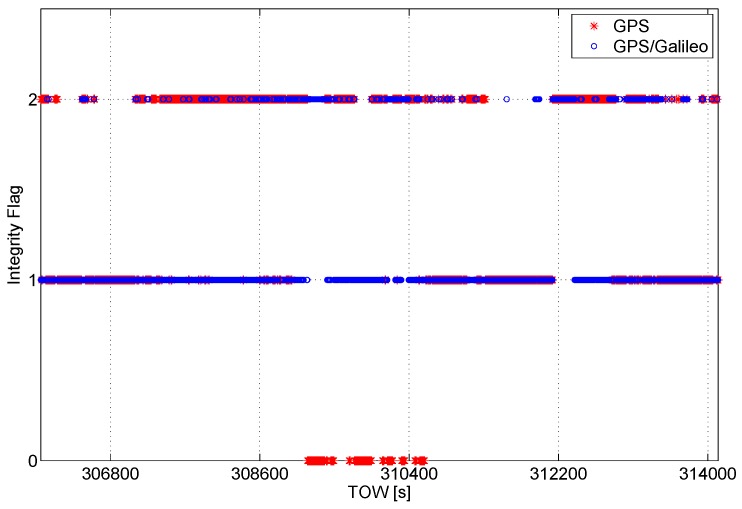
Integrity flag as a function of time. The flag is set to zero when it is not possible to test the reliability of the solution, and one implies a reliable solution, while two indicates an unreliable solution.

The integrity algorithm output is a flag indicating the status of the solution; specifically, if the solution is declared reliable, the flag is set to one. If there is no redundancy to apply the RAIM algorithm or if the geometry conditions are not satisfied, the flag is set to zero. Finally, if the solution is declared unreliable, the flag is set to two. The flag values are plotted as a function of time in Figure 6. Only during a short period of time, the solution is not available for the GPS-only case.

**Figure 7:**
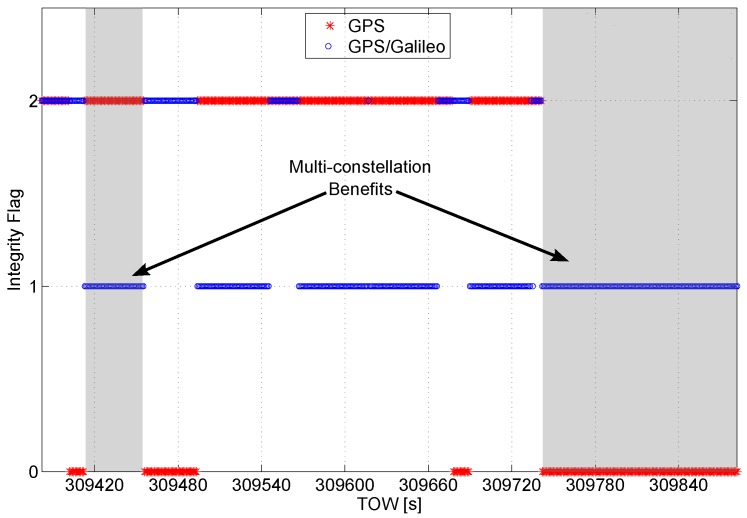
Integrity flag as a function of time. A short interval of the data is considered to better analyze the benefits of the multi-constellation.

In Figure 7, a short time interval of the data collection is considered, and the values of the flag are plotted as a function of time. The benefits of the multi-constellation approach clearly emerge from the figure: in the grey box on the left, the solution passes from unreliable, for the GPS only case, to reliable for the multi-constellation case. In the box on the right, the solution passes from unavailable to reliable.

**Figure 8:**
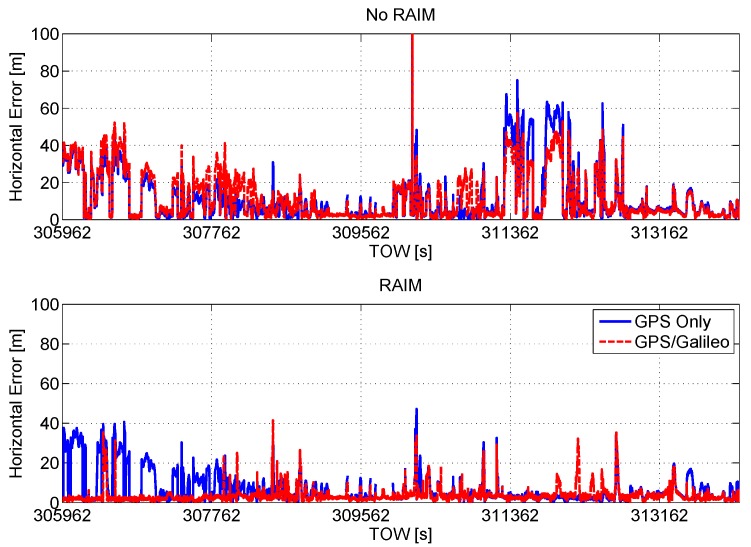
Horizontal error as a function of time. Upper box: horizontal position errors of the configuration without RAIM application; lower box: horizontal position errors of the configurations with RAIM.

The horizontal position errors are plotted as a function of time in Figure 8. In the upper box, the error of the configuration without RAIM application is shown. The presence of gross error in the position solution emerges from the figure: the inclusion of the Galileo measurements provides only a slight reduction of the error, and in a few cases, the inclusion of the measurements provided by Galileo satellites degrades the navigation solution. This is due to the additional blunders present in the measurement set. In the lower box, the horizontal position errors of the configurations with RAIM are shown. Furthermore, in this case, the benefits of the multi-constellation approach clearly emerge: the red line is lower than the blue one.

**Figure 9:**
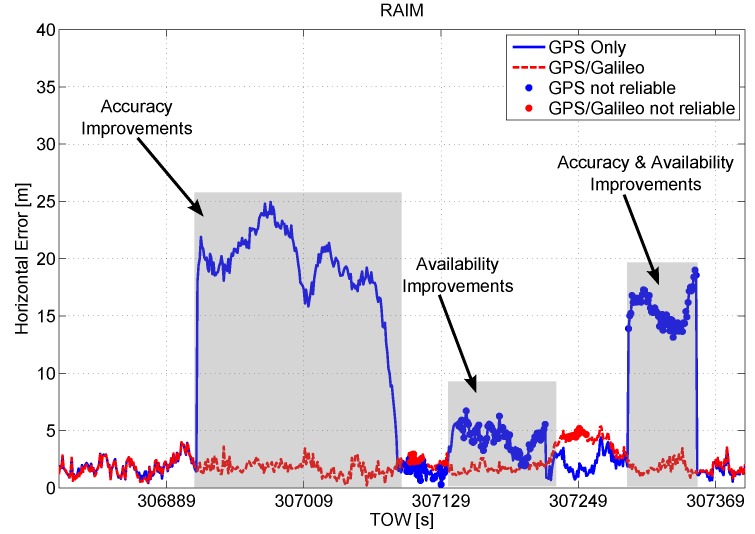
Horizontal error as a function of time. A short time interval of the test is considered to investigate the performance of the configurations with RAIM application.

In order to further analyze the performance of the configurations with RAIM, a short time interval of the test is considered in Figure 9, where the horizontal position errors are plotted as a function of time. The synergy between the GPS/Galileo multi-constellation solution and the implemented RAIM algorithm provides clear benefits. In particular, in the first grey box, the introduction of the Galileo measurements allows the proper identification of blunders within the GPS measurements improving the accuracy of the navigation solution. From the central grey box, improvements in terms of RA can be noted. Finally, in the grey box on the right, the navigation accuracy is improved and the solution passes from unreliable to reliable when Galileo measurements are included.

**Figure 10:**
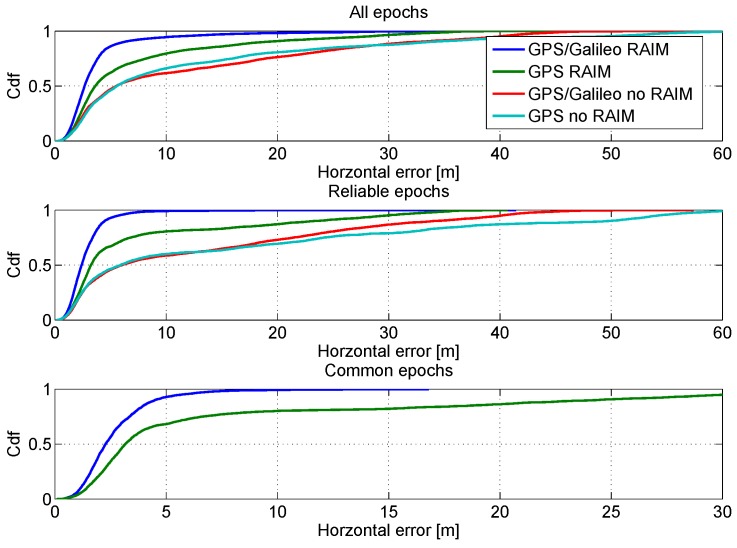
CDFs of the horizontal errors. Upper box: CDFs of the horizontal position errors computed using all epochs; central box: CDFs computed using only the reliable epochs; lower box: CDFs computed using only common epochs.

In order to have a more clear representation of the performance in the horizontal channel, the CDFs of the horizontal errors are shown in Figure 10. In the upper box, the CDFs of the horizontal position errors are computed using all the epochs; the impact of the RAIM algorithm clearly emerges from the figure: the blue and green lines are higher than the other lines. The advantages are more evident in the central box, where the CDFs are computed using only the reliable epochs. Finally, in the lower box of Figure 10 the comparison between GPS and GPS/Galileo multi-constellation is performed using only the common epochs. The benefits of the introduction of the Galileo measurements clearly emerge from the figure: the blue line is higher than the green one.

**Figure 11:**
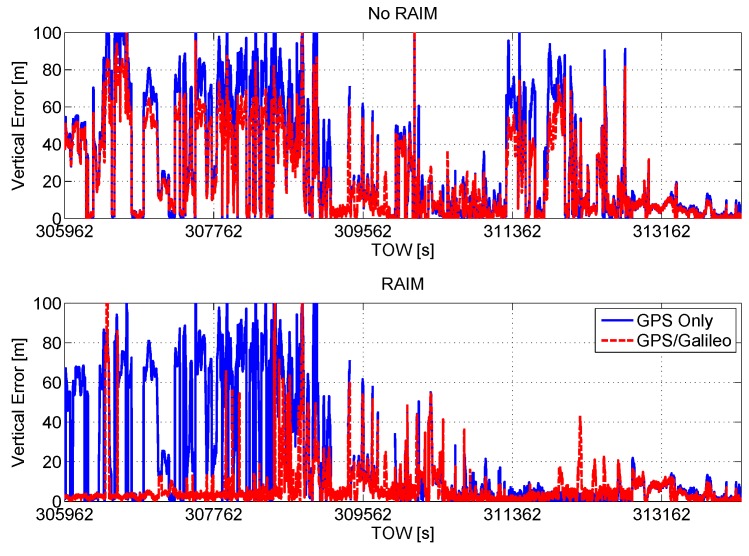
Vertical position error as a function of time. Upper box: vertical errors of the configuration without RAIM application; lower box: vertical errors of the configurations with RAIM.

In Figure 11, the vertical position errors are plotted as a function of time. In the upper box of the figure, the vertical errors of the configurations without RAIM are plotted; the introduction of the Galileo measurements without quality control provides only a slight improvement, since the blue and red lines in Figure 11 are very close. In the lower box of Figure 11, the vertical position errors of the configurations with the RAIM application are shown. The inclusion of Galileo observables and the use of the RAIM technique have a clear impact on the system performance. GPS/Galileo multi-constellation solutions are characterized by significantly reduced errors.

**Figure 12:**
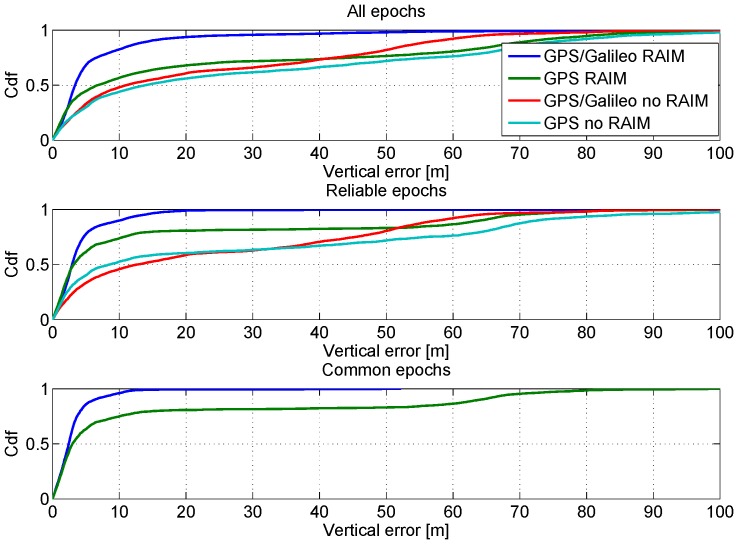
CDFs of the vertical position errors. Upper box: CDFs of the vertical errors computed using all epochs; central box: CDFs computed using only the reliable epochs; lower box: CDFs computed using only common epochs.

The CDFs of the vertical position errors of the four configurations considered are shown in Figure 12. In the upper box of the figure, the CDFs are computed using all available epochs: three of the four configurations, *i.e.*, GPS noRAIM, GPS RAIM and GG noRAIM, have similar performance with only a slight improvement in the GPS RAIM case. However, the GPS/Galileo multi-constellation solution with RAIM application provides the best solution by far. A little improvement in the performance of the GPS RAIM configuration is obtained when the CDF is computed using only reliable epochs, as shown in the central box of Figure 12. In order to have a fair comparison between GPS/Galileo and GPS only solutions, the CDFs are computed using only the reliable epochs in the lower box of Figure 12. The enhancements due to the Galileo measurements clearly emerge from the comparison.

**Figure 13:**
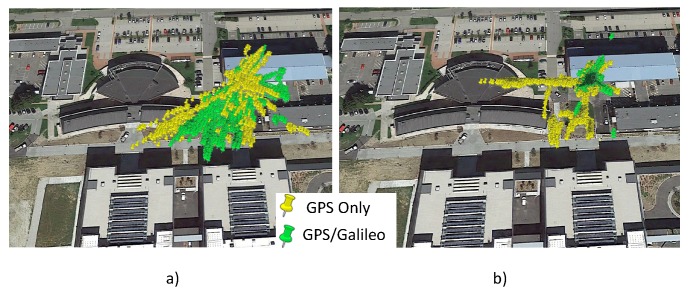
Horizontal position solutions computed using the configurations analyzed. (**a**) Position solutions computed using the configurations without RAIM; (**b**) position solutions computed using the configurations with RAIM.

The horizontal position scatter plots of the configurations analyzed are shown in Figure 13. In Figure 13a, the dispersion of the configurations without RAIM are plotted, whereas the horizontal positions provided by the configurations with the RAIM application are shown in Figure 13b. From the figures, the improvements of the RAIM application are evident: the clouds in Figure 13b are more concentrated than the ones in Figure 13a. The benefits of the Galileo measurements’ introduction are even more clear: in both cases, the green clouds are more concentrated than in the yellow ones.

The parameters used to summarize the performance of the configurations considered are the maximum, mean and SD of the horizontal and vertical errors. The parameters are computed for the four configurations and are reported in Table 3, Table 4 and Table 5. In Table 3, the parameters are computed using all available epochs; in Table 4, only reliable epochs are considered; and finally, in Table 5, only common reliable epochs are used. In all cases, the synergy between GPS/Galileo and the RAIM algorithm achieves the best performance with the lowest error values.

**Table 3:** Position error statistics considering the different solutions and all epochs.

Configuration	Max H (m)	Max up (m)	SD H (m)	SD up (m)	Mean H (m)	Mean up (m)
GPS noRAIM	400.02	514.23	16.02	32.32	12.09	29.70
GG noRAIM	387.12	542.52	14.33	26.59	12.08	−21.12
GPS RAIM	47.28	127.89	8.05	29.06	7.10	22.91
GG RAIM	41.47	143.43	4.15	13.06	3.74	−4.75

**Table 4:** Position error statistics considering only reliable epochs.

Configuration	Max H (m)	Max up (m)	SD H (m)	SD up (m)	Mean H (m)	Mean up (m)
GPS noRAIM	75.10	123.00	17.65	31.32	15.44	27.27
GG noRAIM	57.40	101.57	12.90	24.93	12.72	22.22
GPS RAIM	40.71	106.96	8.94	24.52	7.25	15.08
GG RAIM	41.44	125.49	2.00	7.35	2.78	−2.86

**Table 5:** Position error statistics considering only common reliable epochs.

Configuration	Max H (m)	Max up (m)	SD H (m)	SD up (m)	Mean H (m)	Mean up (m)
GPS noRAIM	75.10	123.01	17.46	31.18	15.38	26.93
GG noRAIM	57.40	101.57	14.79	25.61	14.84	−19.84
GPS RAIM	40.71	106.96	9.19	24.46	7.36	14.89
GG RAIM	16.80	52.17	1.67	4.91	2.68	−1.41

### 5.2. Velocity Results

In this section, the results obtained in the velocity domain are presented.

**Figure 14:**
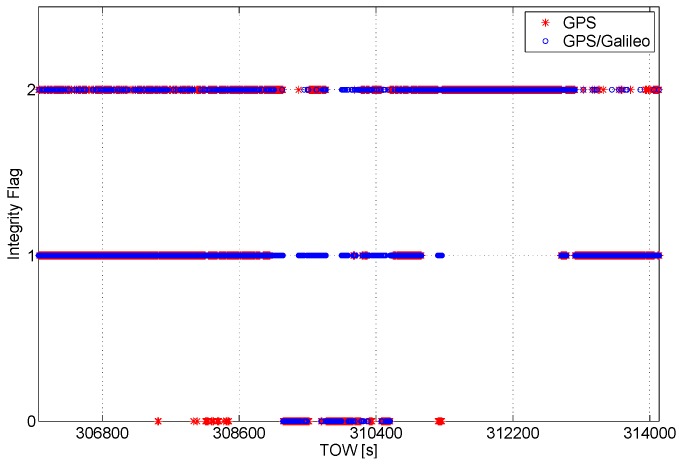
Integrity flag as a function of time. The flag is set to zero when it is not possible to test the reliability of the solution; one implies a reliable solution; two indicates an unreliable solution.

**Figure 15:**
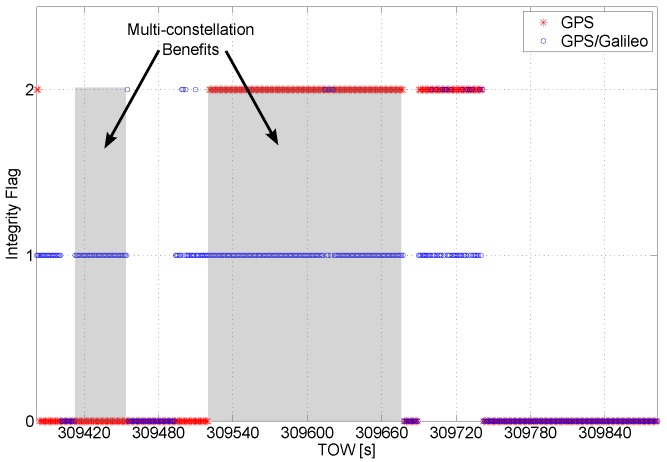
Details of the velocity integrity flag around epochs where the GPS-only solution is declared unreliable.

The integrity algorithm is applied to Doppler shift measurements in order to identify blunders that can degrade the velocity solution. One of the outputs is the flag indicating the status of the solution. The integrity flag related to the velocity solutions is plotted as a function of time in Figure 14. A short time interval of the dataset is analyzed in Figure 15, which better highlights the benefits of the multi-constellation solution.

Furthermore, in the velocity domain, data are characterized by a high SA, which is 95% in the case of GPS only: the inclusion of the Galileo measurements improves the SA by about 5%. The application of the RAIM algorithm reduces the availability of the solution, which in the GPS only case is more than halved passing from 95% to 45%; in this case, the benefit of the introduction of the Galileo observables is more evident with an enhancement of 23% with respect to the GPS-only case. The results related to the SA and RA of the velocity solution are detailed in Table 6.

**Table 6:** SA and RA of the velocity solutions.

Configuration	SA (%)	RA (%)
GPS noRAIM	95	N.A.
GG noRAIM	100	N.A.
GPS RAIM	95	45
GG RAIM	100	62

**Figure 16:**
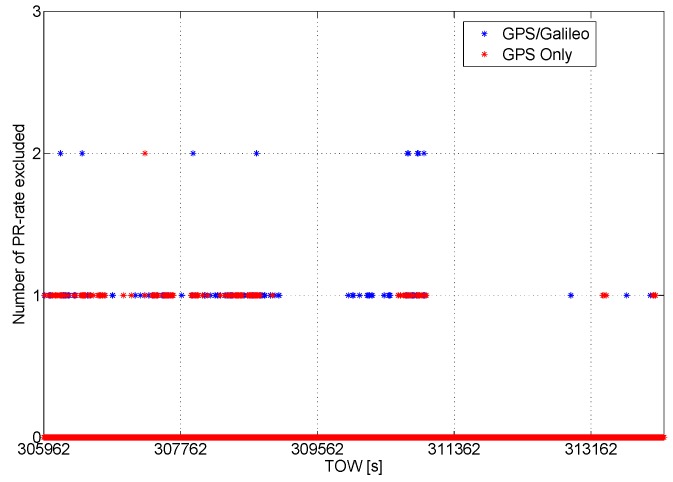
Number of pseudorange-rate observables excluded from the navigation solution as a function of time.

The number of pseudorange-rate measurements excluded from the navigation solution is plotted as a function of time in Figure 16: the number of exclusions is lower than in the pseudorange case; however, also in this case, the introduction of the Galileo measurements improves the redundancy of the system, enhancing the performance of the RAIM algorithm, which is able to reject a higher number of observables.

**Figure 17:**
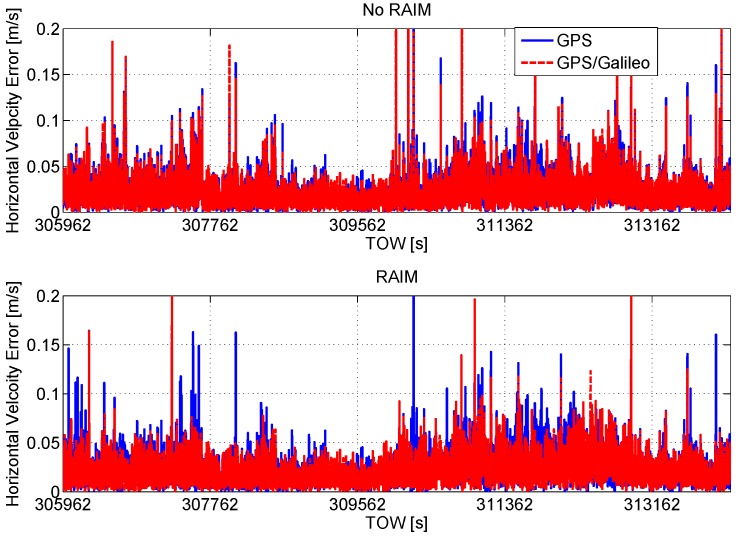
Horizontal velocity errors as a function of time. Upper box: configurations without RAIM; lower box: configurations with RAIM.

The horizontal velocity errors are plotted as a function of time in Figure 17. In the upper box of the figure, the configurations without RAIM show similar performance: the introduction of the Galileo measurements provides only a slight error reduction. In the lower box, where the configurations without RAIM are considered, the benefits of the Galileo inclusion are more evident; the use of RAIM improves the performance mainly in terms of maximum errors, which are strongly reduced. The statistical parameters of the velocity errors considering all of the solutions are summarized in Table 7.

**Table 7:** Velocity error statistics considering all epochs.

Configuration	Max H (m/s)	Max up (m/s)	SD H (m/s)	SD up (m/s)	Mean H (m/s)	Mean up (m/s)
GPS noRAIM	1.686	2.548	0.037	0.069	0.025	−0.001
GG noRAIM	1.680	2.566	0.043	0.065	0.023	−0.001
GPS RAIM	0.252	0.411	0.017	0.048	0.024	0.003
GG RAIM	0.256	0.244	0.015	0.043	0.021	0.001

**Figure 18:**
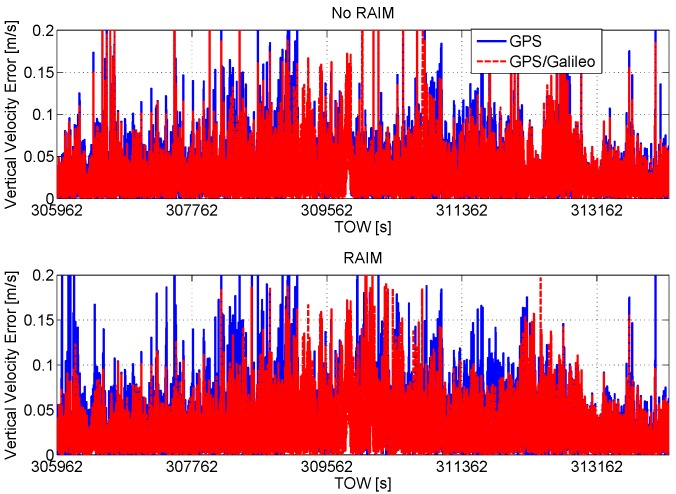
Vertical velocity errors as a function of time. Upper box: configurations without RAIM; lower box: configurations with RAIM.

The vertical velocity errors are plotted as a function of time in Figure 18: the configurations without RAIM application are nearly coincident with a maximum error that exceeds 2 m/s. The RAIM algorithm reduces all of the error parameters, in particular the maximum errors are reduced by about five times. In the vertical case, the benefit of the Galileo inclusion is more evident than in the horizontal case.

**Figure 19:**
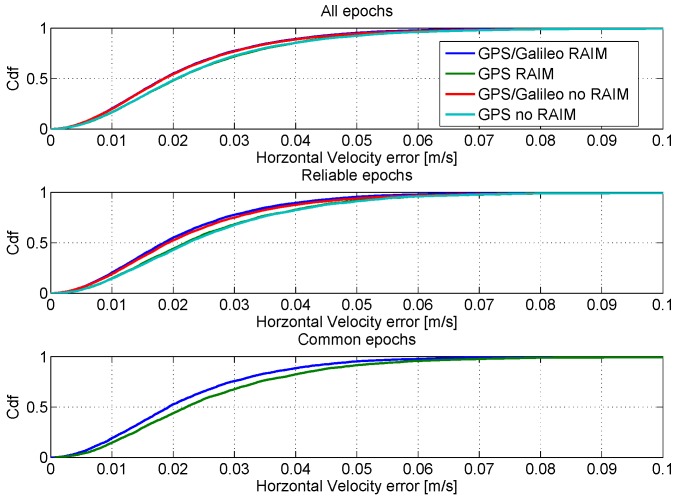
CDFs of the horizontal velocity errors. Upper box: CDFs computed using all available epochs; central box: CDFs computed using only reliable epochs; lower box: CDFs computed using only reliable common epochs.

**Figure 20:**
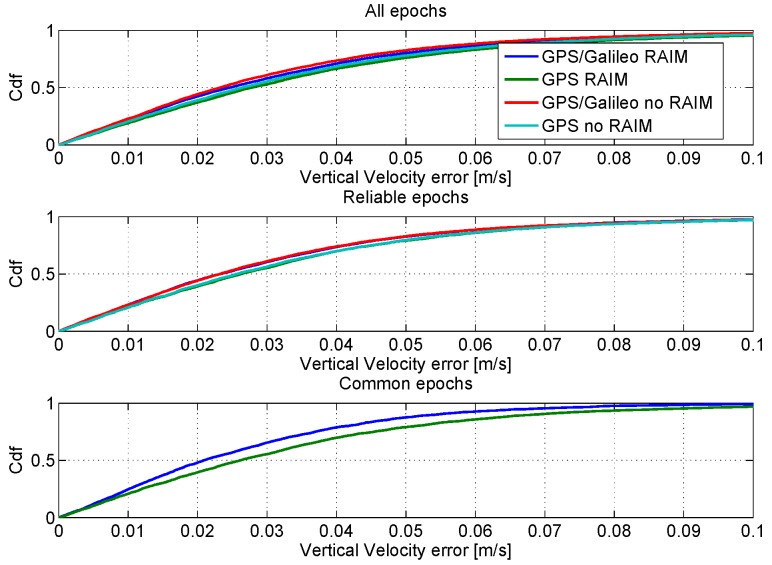
CDFs of the vertical velocity errors. Upper box: CDFs computed using all available epochs; central box: CDFs computed using only reliable epochs; lower box: CDFs computed using only reliable common epochs.

In order to have a fair comparison among the considered configurations, the CDFs of the horizontal and vertical velocity errors are shown in Figure 19 and Figure 20, respectively. As for the position domain, three different time frames have been identified and used to compute the CDFs. In particular, the CDFs computed using all of the available epochs are shown in the upper boxes of the figures. In the central boxes, the CDFs using only reliable epochs are shown, and finally, in the lower boxes, the CDFs using only the common reliable epochs are provided. The figures show that all of the configurations have similar performance in the velocity domain: only a slight improvement can be appreciated using the GPS/Galileo multi-constellation solution together with the application of the RAIM algorithm. 

Statistical error parameters are computed using only reliable epochs in Table 8: the main advantage of RAIM application is the reduction of the maximum errors in both the horizontal and vertical channel. The maximum horizontal velocity error passes from 0.45 m/s, in the GPS-only case without RAIM, to 0.16 m/s applying the RAIM algorithm. The improvements in the multi-constellation case are even more evident: the maximum error is reduced almost six times, passing from 1.68 m/s to 0.24 m/s.

**Table 8:** Velocity error statistics considering only reliable epochs.

Configuration	Max H (m/s)	Max up (m/s)	SD H (m/s)	SD up (m/s)	Mean H (m/s)	Mean up (m/s)
GPS noRAIM	0.453	0.464	0.017	0.039	0.022	0.010
GG noRAIM	1.680	0.403	0.028	0.037	0.021	0.004
GPS RAIM	0.163	0.318	0.014	0.037	0.021	0.010
GG RAIM	0.239	0.187	0.012	0.035	0.019	0.004

Table 9 further investigates the velocity error statistics where only reliable epochs common to both GPS-only and GPS/Galileo configurations are considered. The results support the previous findings. Moreover, it is shown that, in certain cases, the inclusion of Galileo measurements can degrade the velocity solution. RAIM is required to screen out bad measurements possibly introduced by an additional constellation.

**Table 9:** Velocity error statistics considering only common reliable epochs.

Configuration	Max H (m/s)	Max up (m/s)	SD H (m/s)	SD up (m/s)	Mean H (m/s)	Mean up (m/s)
GPS noRAIM	0.453	0.464	0.016	0.038	0.021	0.010
GG noRAIM	1.680	0.403	0.032	0.034	0.021	0.008
GPS RAIM	0.149	0.318	0.013	0.036	0.021	0.010
GG RAIM	0.092	0.183	0.011	0.030	0.018	0.008

### 5.3. Comparison with Respect to the Classical RAIM Algorithm

In order to analyze the benefits of the additional checks introduced, the proposed algorithm and the classical RAIM algorithm are compared considering GPS and Galileo measurements. Classical RAIM does not have geometry and separability tests and performs only one exclusion.

**Figure 21:**
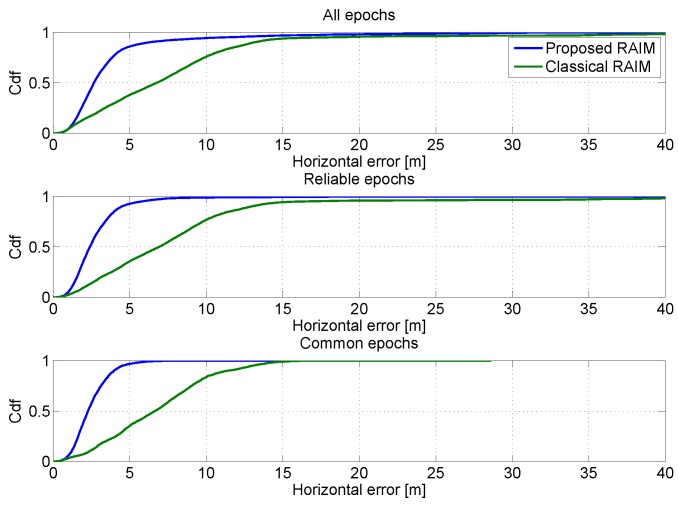
CDFs of the horizontal position errors when a classical RAIM approach is considered. Upper box: CDFs computed using all available epochs; central box: CDFs computed using only reliable epochs; lower box: CDFs computed using only reliable common epochs.

The CDFs of the horizontal errors obtained in the two cases are plotted in Figure 21. As for the previous cases, the CDFs considering all available epochs is shown in the upper box of the figure; in the central box, the performance of the considered configurations is obtained considering only reliable epochs. Finally, in the lower box, the CDFs are computed using only reliable common reliable epochs. In all cases, the advantages of the tests introduced can be clearly appreciated: the blue lines are higher than the green ones.

**Figure 22:**
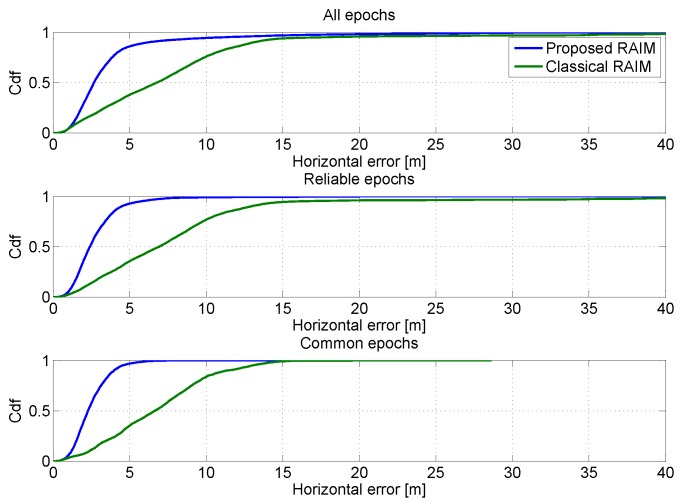
CDFs of the vertical position errors when a classical RAIM approach is considered. Upper box: CDFs computed using all available epochs; central box: CDFs computed using only reliable epochs; lower box: CDFs computed using only reliable common epochs.

In Figure 22, the CDFs of the vertical position errors considering three different time frames are plotted. The improvements due to the geometry and separability tests are evident: also in this case, the proposed algorithm provides reduced errors with respect to the classical RAIM algorithm. The performance of the configurations analyzed is summarized in Table 10, Table 11 and Table 12.

**Table 10:** Position error statistics considering the different solutions and all epochs. Classical RAIM is used as the term of comparison.

Configuration	Max H (m)	Max up (m)	SD H (m)	SD up (m)	Mean H (m)	Mean up (m)
Classical RAIM	74.45	164.76	7.62	20.35	7.94	−5.55
Proposed algorithm	41.47	143.43	4.15	13.06	3.74	−4.75

**Table 11:** Position error statistics considering only reliable epochs. Classical RAIM is used as the term of comparison.

Configuration	Max H (m)	Max up (m)	SD H (m)	SD up (m)	Mean H (m)	Mean up (m)
Classical RAIM	32.18	24.29	3.47	5.55	6.80	−2.29
Proposed algorithm	41.44	125.49	2.00	7.35	2.78	−2.86

**Table 12:** Position error statistics considering only common reliable epochs. Classical RAIM is used as the term of comparison.

Configuration	Max H (m)	Max up (m)	SD H (m)	SD up (m)	Mean H (m)	Mean up (m)
Classical RAIM	28.63	24.29	3.48	5.45	6.72	−2.54
Proposed algorithm	19.02	20.74	1.18	3.70	2.47	−2.30

The benefits of the proposed algorithm clearly emerge from the tables. When all epochs are considered, the proposed algorithm provides improved performance with respect to the classical RAIM algorithm. The improvements are evident for all of the considered parameters, *i.e.*, the maximum and mean error and the error SD. The improvement is present for both horizontal and vertical components. When only reliable epochs are considered, the classical RAIM algorithm provides better performance in terms of maximum horizontal and vertical errors. However, in this case, the performance of the two configurations is evaluated considering different epochs, because the algorithm provides different RA, which in the case of the classical algorithm is 42.33% (78% was obtained with the approach proposed). The classical RAIM approach is more conservative and does not foresee the possibility of having multiple exclusions. In order to perform a fair comparison between the considered configurations, the performance is evaluated considering only common reliable epochs in Table 12: the proposed algorithm reduces all of the statistical error parameters considered. 

## 6. Conclusions

In this work, an effective RAIM algorithm for LBS applications has been implemented. The algorithm exploits the potential of a GPS/Galileo multi-constellation solution. An RAIM quality check for GPS/Galileo multi-constellation navigation has been considered and experimentally analyzed considering a harsh environment where a single GNSS may not satisfy minimum navigation requirements in terms of availability, continuity, accuracy and integrity. The parameters adopted to evaluate the performance of the algorithm in terms of accuracy were the SD, maximum and mean errors. The parameters were computed for both horizontal and vertical components of the position and velocity solutions.

In order to evaluate the performance in terms of availability, two different parameters were adopted depending on the application or not of RAIM techniques. Specifically, the SA is used when the RAIM algorithm is not applied, and the RA is introduced when quality checking is performed. In order to assess the performance of the configurations considered, a static test has been carried out: data were collected in a parking area between three high buildings, which reflect GNSS signals, causing severe multipath errors in the GNSS measurements. Hence, the assumption of a single blunder within the measurement set is not realistic. For this reason, quality checking has been performed iteratively, rejecting observations until no additional outliers are identified or until the solution is declared impossible to check. The classical FB algorithm has been modified, introducing a preliminary check to verify the robustness of the satellite geometry and a separability check to avoid erroneous rejection. The algorithm has been further modified considering different weights for GPS and Galileo observables.

From the analysis, it emerges that GPS alone is not able to provide accurate and continuous navigation in signal-degraded environments. Although the introduction of RAIM improves the performance of GPS-only navigation, the solution availability is significantly reduced. This availability reduction is compensated by the inclusion of Galileo measurements, which enhance performance with respect to the baseline configuration. The inclusion of Galileo measurements alone may not be sufficient for achieving the required level of performance, since also the multi-constellation solution is characterized by large errors. The joint adoption of Galileo and RAIM improves performance with respect to the baseline configurations for all of the considered parameters: horizontal error parameters are more than halved with respect to configurations without RAIM. Furthermore, the vertical errors are significantly reduced.

The performance of the proposed algorithm is compared with respect to the performance of the classical RAIM algorithm: the proposed approach provides improvements in terms of maximum and mean errors for both horizontal and vertical components.

In the velocity domain, the benefits of the Galileo inclusion are less evident: the combined use of Galileo observables and RAIM algorithms improves the performance mainly in terms of maximum errors, which are strongly reduced in both horizontal and vertical components.

Finally, the performance of the proposed algorithm is compared to that obtained using the classical FB algorithm: the benefits of the proposed modification clearly emerge. The proposed algorithm provides improvements for all of the considered parameters.

With this in mind, the synergy between multi-constellation solutions and RAIM can be a key element for enabling LBSs in signal-degraded environments.
